# Mirage 2.0: fast and memory-efficient reconstruction of gene-content evolution considering heterogeneous evolutionary patterns among gene families

**DOI:** 10.1093/bioinformatics/btac433

**Published:** 2022-06-30

**Authors:** Tsukasa Fukunaga, Wataru Iwasaki

**Affiliations:** Waseda Institute for Advanced Study, Waseda University, Tokyo 1690051, Japan; Department of Integrated Biosciences, Graduate School of Frontier Sciences, The University of Tokyo, Chiba 2770882, Japan

## Abstract

**Summary:**

We present Mirage 2.0, which accurately estimates gene-content evolutionary history by considering heterogeneous evolutionary patterns among gene families. Notably, we introduce a deterministic pattern mixture model, which makes Mirage substantially faster and more memory-efficient to be applicable to large datasets with thousands of genomes.

**Availability and implementation:**

The source code is freely available at https://github.com/fukunagatsu/Mirage.

**Supplementary information:**

Supplementary data are available at *Bioinformatics* online.

## 1 Introduction

Computational reconstruction of gene-content evolutionary history is a fundamental approach to elucidate the evolution of genomes and biological systems and to estimate functions of function-unknown genes (Fukunaga and Iwasaki, 2022). An essential factor in mathematically modeling gene-content evolution is substantial differences among evolutionary patterns of different gene families (Mendes *et al.*, 2021). For example, immune-related genes are much more likely to be lost from a mammal genome than housekeeping genes (Demuth *et al.*, 2006).

We previously developed Mirage, which reconstructs gene-content evolutionary history with a pattern mixture (PM) model that considers heterogeneous evolutionary patterns among gene families (Fukunaga and Iwasaki, 2021). In this previous study, gene families were probabilistically classified into multiple clusters, each of which follows a specific evolutionary pattern. Although this model [a probabilistic PM (PPM) model] showed high accuracy and fitted empirical data well, it required significant computational time and memory to be readily applicable to datasets with >1000 genomes. Here, we propose a deterministic PM (DPM) model, which is implemented into Mirage 2.0. The DPM model is as accurate as the PPM model, but much faster and more memory-efficient to be applicable to large datasets with ∼3000 genomes and >150 000 OGs in a few days.

## 2 Materials and methods

Given *N* genomes, assume that *M* gene families and a phylogenetic tree *T* are calculated. In the PM model, *M* gene families are classified into *K* clusters, where *K* is a user-specified parameter. Different clusters follow different evolutionary patterns that are represented by cluster-specific transition rate matrices.

Mirage first estimates model parameters using the EM algorithm. The model parameters consist of the cluster-specific transition rate matrices, mixture probabilities of every cluster and cluster-specific copy-number distributions at the root node. Then, Mirage infers gene-content evolutionary history by a Viterbi-like algorithm using the estimated parameters.

The EM algorithm consists of four steps. Step 1: The model parameters are randomly initialized. Step 2: Responsibilities and sufficient statistics of the phylogenetic tree model are calculated based on the model parameters, where a responsibility is defined as a probability of assigning each gene family to each cluster. In this model, the sufficient statistics are expected duration of each copy number and expected numbers of copy number changes across the tree (Kiryu, 2011). Step 3: The model parameters are estimated and updated by assuming those responsibilities and sufficient statistics. Step 4: Steps 2 and 3 are repeated until the log-likelihood given by the estimated parameters converges. In Step 2, the inside algorithm, responsibility calculation, outside algorithm and sufficient statistics calculation are conducted in this order. The inside and outside algorithms calculate the inside and outside values for each combination of nodes in the phylogenetic tree, clusters and gene families. For each node and gene family, the inside value is defined as the probability of the descendant nodes given the state of the node and the estimated parameters. The outside value is defined as the joint probability of the non-descendant nodes and the state of the node given the estimated parameters. Responsibilities are calculated based on the inside values of the root node, and sufficient statistics are calculated based on both the inside and outside values.

In the PPM model, the responsibilities take real numbers between 0 and 1, i.e. each gene family can be assigned to all clusters with non-zero positive probabilistic weights even if the weights are very small. This resulted in unnecessarily large computational cost in Step 2 of the EM algorithm. In the DPM model, the responsibilities take 1 for one cluster and 0 for the others. Specifically, after the calculation of the responsibilities, the responsibility of a cluster with the largest responsibility and those of the other clusters are set to 1 and 0, respectively, for each gene family. Calculation of outside values and sufficient statistics is skipped if responsibilities are 0, substantially reducing computation time and memory in the outside algorithm and sufficient statistics calculation in Step 2 of the EM algorithm. We note that the DPM model is similar to partitioning methods in molecular evolution (Lanfear *et al.*, 2017) and a classification EM algorithm in machine learning (Celeux and Govaert, 1992).

Another major update in Mirage 2.0 is in the calculation method of sufficient statistics. The previous version calculated integrals of matrix exponential by an eigen decomposition method, which cannot be applied to non-diagonalizable matrices and can result in unstable learning. Mirage 2.0 uses the auxiliary matrix method, which does not have this drawback (Liu *et al.*, 2015).

## 3 Results

We evaluated Mirage based on the PPM and DPM models using three previously constructed empirical datasets (Fukunaga and Iwasaki, 2021). The fungi, archaea and micrococcales datasets contained 123 genomes and 34 454 OGs, 151 genomes and 11 650 OGs and 111 genomes and 9523 OGs, respectively.

**Fig. 1.**
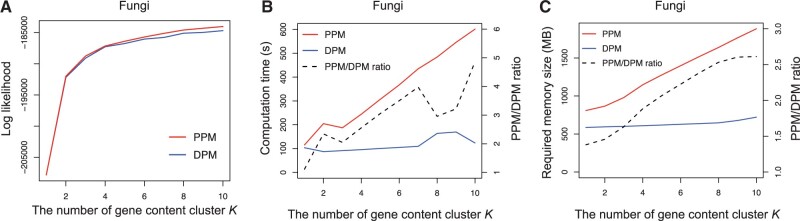
Mirage with the PPM and DPM models on 123 fungal genomes. Results by the PPM and DPM models are represented by red and blue lines, respectively. Black dashed lines represent their ratios. (**A**) Log-likelihood values in hold-out validation experiments. (**B**) Computational time. (**C**) Required memory size (A color version of this figure appears in the online version of this article.)

Performance of gene-content evolutionary history estimation was evaluated for each dataset by log-likelihood values. Hold-out validation experiments were performed by splitting the OGs into training and test datasets at a 4:1 ratio. The model parameters were estimated using the training dataset 100 times and the best parameters were chosen. Log-likelihood was calculated by applying the best parameters to the test dataset. The PPM and DPM models showed comparable log-likelihood values in all datasets (Fig. 1A and Supplementary Fig. S1A and B).

Computational time and memory required for 10 EM steps were measured on Intel Xeon Gold 6130 2.1 GHz CPU with 4 GB memory, and average values of 100 measurements were compared for each dataset (Fig. 1B and C and Supplementary Fig. S1C–F). While the required time and memory almost linearly increased with *K* in the PPM model, they were almost independent of *K* in the DPM model. Therefore, in terms of speed and memory, the DPM model becomes substantially more efficient when *K* > 5. It would be notable that it is quite common to classify OGs into such numbers of clusters (e.g. COGs have >20 categories).

Numbers of EM-step iterations were also compared by setting *K* = 5, because more iterations would result in slower convergence. For each measurement using each dataset, the PPM and DPM models were run with the same seed values for randomization. Based on 100 measurements, we confirmed that the DPM model has significantly smaller numbers of EM-step iterations than the PPM model (Wilcoxson signed-rank test, Supplementary Figs S2 and S3). Somewhat interestingly, we did not find correlations between the iteration numbers by the PPM and DPM models with the seed values.

Finally, to demonstrate that the DPM model is now applicable to a large dataset with reasonable computational resources, we reconstructed the gene-content evolutionary history of the whole bacterial domain with *K* = 10. The computation was conducted on Intel Xeon Gold 6154 4.0 GHz CPU with 128 GB memory. Pre-processing OGs from STRING database version 11.5 (Szklarczyk *et al.*, 2021) and a phylogenetic tree from GTDB release 202 (Parks *et al.*, 2022) as previously described (Fukunaga and Iwasaki, 2021) produced 2 848 genomes and 169 170 OGs. With this data, while the PPM model was not able to run due to insufficient memory, the DPM model successfully reconstructed its whole gene-content evolutionary history with 42.94 h and only 37.9 GB memory (average of 100 runs).

## Supplementary Material

btac433_Supplementary_Data
