# Supplementary material for: Mirage 2.0: fast and memory-efficient reconstruction of gene-content evolution considering heterogeneous evolutionary patterns among gene families
Source: Bioinformatics. 2022 Jun 30;38(16):4039–41. doi: 10.1093/bioinformatics/btac433 (PMC9364385; doi:10.1093/bioinformatics/btac433)
Supplement: btac433_Supplementary_Data [file btac433_supplementary_data.pdf]

Supplementary Materials for Mirage 2.0: fast and memory-efficient reconstruction of gene-content evolution considering heterogeneous evolutionary patterns among gene families

Tsukasa Fukunaga and Wataru Iwasaki

Supplementary Figures

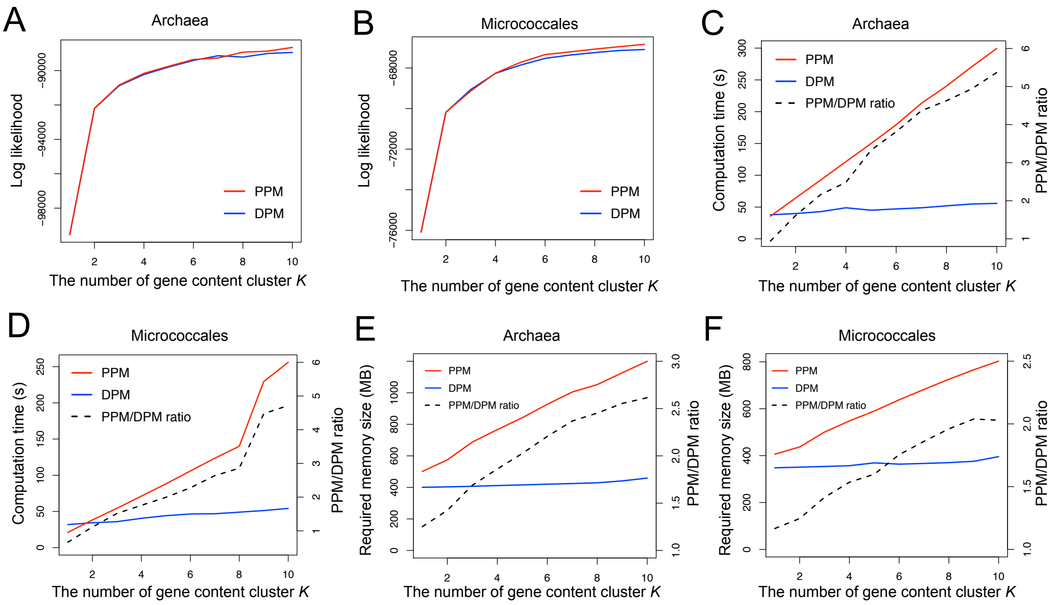

Fig. S1 The results of comparative analysis between the PPM and DPM models for the archaea and micrococcales dataset. The PPM and DPM models are represented by solid red and blue lines, respectively. Black dashed lines represent the ratio of the PPM model to the DPM model. The x-axes represent the number of gene content clusters K. (A-B) Log-likelihood values in the holdout validation experiments. The y-axis represents the log-likelihood values. (C-D) The results of the computational time evaluation. The left and right y-axes represent the computational time (s) and the PPM/DPM ratio, respectively. (E-F) The results of the evaluation of the required memory size. The left and right y-axes represent the required memory size (MB) and the PPM/DPM ratio, respectively. The panels of (A), (C) and (E) represent the results of archaea dataset analysis, and those of (B), (D) and (F) represent the results of micrococcales dataset analysis,

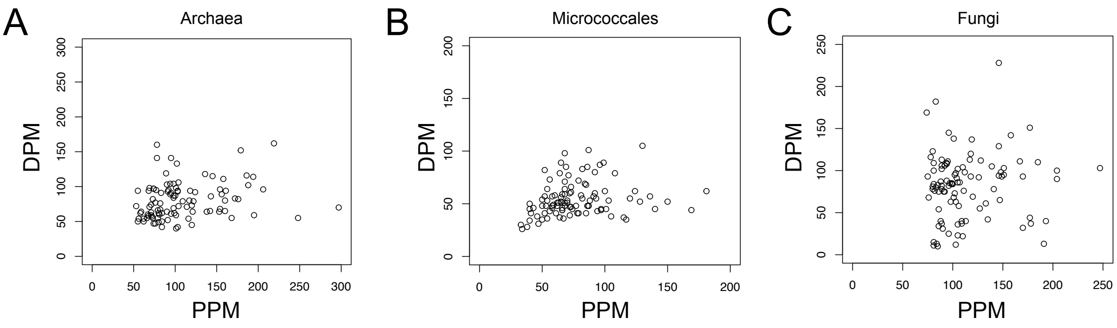

Fig. S2 The correlation of the numbers of iterations in the EM algorithm between the PPM and DPM model. The x-axes and the y-axes represent the PPM and DPM model, respectively. The panels represent the results for (A) the archaea dataset, (B) the micrococcales dataset, and (C) the fungi dataset.

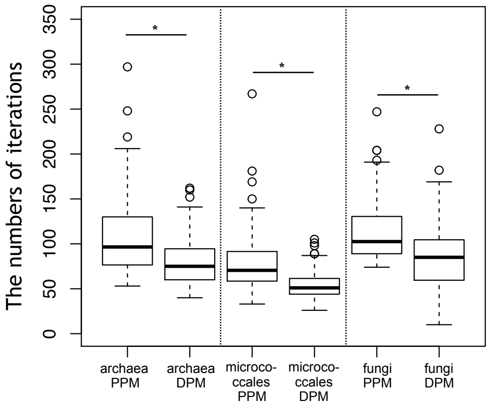

Fig. S3 The comparison of the numbers of iterations in the EM algorithm between the PPM and DPM model. The y-axis represents the numbers of iterations. * means the statistical significance under the Wilcoxson signed-rank test.
